# Evaluating the impact of soil erosion on soil quality in an agricultural land, northeastern China

**DOI:** 10.1038/s41598-024-65646-5

**Published:** 2024-07-07

**Authors:** Haiyan Fang, Yuyu Zhai, Chaoyue Li

**Affiliations:** 1 Key Laboratory of Water Cycle and Related Land Surface Processes, Institute of Geographic Sciences and Natural Resources Research, Chinese Academy of Sciences, Beijing, 100101 China; 2 College of Resources and Environment, University of Chinese Academy of Sciences, Beijing, 100049 China

**Keywords:** Soil erosion, Soil quality, TDS, MDS, Topography, Environmental impact, Hydrology

## Abstract

The impact of soil erosion on soil quality is still not systematically understood. The purpose of this study was thus to quantify the impact of soil erosion on soil quality and its change with slope morphology in an agricultural field, northeastern China based on radionuclide ^137^Cs, unmanned aerial vehicle derived high resolution digital elevation model, and soil sampling. ^137^Cs method yielded an average soil erosion rate of − 275 t km^−2^ yr^−1^ ranging from − 1870 to 1557 t km^−2^ yr^−1^. The soil quality index derived from total dataset (SQI_TDS) can be well explained by that derived from minimum data set (SQI_MDS) with a determination coefficient R^2^ of 0.874. SOM, sand, and cation exchange capacity in the MDS play more important roles than other soil indicators. Soil quality was significantly affected by soil erosion, with Adj. R^2^ of 0.29 and 0.33 for SQI_TDS and SQI_MDS, respectively. The spatial variations of soil erosion and soil quality were both affected by slope topography. Soil erosion must be controlled according to topographic and erosion characteristics in northeastern China.

## Introduction

Soil is an important part of the earth’s biosphere, the depletion of soil nutrients usually leads to land degradation and reduces soil production capacity^1^. In recent years, intensive anthropogenic activities are further accelerating land degradation and decreasing soil quality, especially in agricultural land where human-induced soil erosion is causing losses of nutrients and organic matter^2^, reducing soil depth^3^, decreasing soil microorganisms^4,5^, and even leading to higher concentration of toxins^6^. An accurate evaluation of soil quality and its response to soil erosion is imperative for agricultural production, economic well-being, and government decision making^7,8^.

In recent years, many studies on soil quality have been conducted, and found that soil quality is influenced by a variety of factors including land use, land management, soil erosion and conservation, and morphology^9–12^. However, most of the studies were concentrated on land use and land management factor. For example, Turan et al.^13^ assessed soil quality in the desertification and degradation regions using multivariate and fuzzy methods. Zhang et al.^14^ compared soil qualities with different ecological engineering enclosures in an alpine desert grassland area. Li et al.^15^ compared soil quality in the urban–rural fringe to nearby grain fields, open-air vegetation plots, facility vegetable plots in Daxing District, Beijing, China. Da Rocha Junior et al.^16^ also studied the effect of land use types and landscape position on soil quality in the Alegre region Brazil. Similar studies were also reported in other studies^17–19^. Additionally, soil erosion including rock chemical weathering can also greatly affect carbon emission^15,20–22^, induce soil organic carbon and soil nutrients losses, and then decrease soil quality. How does it influence soil quality, and how does it change with slope morphology? Detailed exploration of these problems is very important for the development of precision agriculture system^23^.

However, most previous studies focus on the effect of land use on soil quality, the impact of soil erosion on soil quality and their responses to land morphology in agricultural lands are still not systematically understood^12^. Soil erosion has been found to be the driving force of most soil quality changes^9,24^. The huge soil loss from poor land/soil management could seriously impact soil quality and sustainable use of soil resource. Mandal et al.^1^ demonstrated that the severe erosion decreases soil quality. This finding was also found by Jin et al.^4^ in the purple soil region in China. Slope morphology is one of the most important factors in influencing soil erosion and soil quality. Pham et al.^25^ studied the impact of topographic aspect on soil quality of agricultural lands in the Hue city in Vietnam. The functional links between land use, geomorphology, soil erosion and soil quality in a catchment in central Iran were also evaluated by Derakhshan-Babaei et al.^12^. Soil erosion on farmland is of great concern and regarded as one of the most serious environmental problems in the world^26^. Cultivation can accelerate soil erosion by reducing soil resilience and clearing aboveground vegetation that is more substantial under frequent climatic extremes^27^. Therefore, around 6.7 million ha of productive land is lost with annual loss rate of 24 million in the world^28^. It is vitally important to study the impact of soil erosion on agricultural soil quality for ecosystem sustainability and land use management^29^. However, high-resolution exploration of the impact of soil erosion on soil quality as well as their variations with slope morphology was less done in agricultural lands.

The black soil region in northeastern China has an area of around 1.24 million square kilometers, and is considered essential for Chinese grain production^30^. Nevertheless, severe soil erosion occurred during the past decades, the thickness of the A-horizon of the black soils has decreased from around 60–70 cm to 20–30 cm in depth, and in some regions, the loess parent material even has been exposed^31,32^. However, few studies on soil quality have been conducted in northeastern China. Some studies evaluated the changes of soil organic carbon induced by soil erosion^33–35^^.^ Studies on soil quality were also conducted in this region. Wang et al.^36^ evaluated soil quality in wetland in the Sanjiang Plain with different methods. Chen et al.^37^ also evaluated soil quality in Hailun County, northeastern China, and correlated soil quality with soil productivity. Li et al.^38^ assessed soil quality of croplands in the black soil zone of Jilin Province, China. Due to soil erosion, the variations of soil productivity were also reported in the black soil regions^39,40^. Unfortunately, these studies even neglected the impact of soil erosion and morphology on soil quality although this region has suffered severe soil erosion. The fluctuated topography in this region greatly influences redistributions of soil as well as soil quality^32^. Deep understanding the patterns of soil erosion and its impact on soil quality in this kind of landscape is very important to implement efficient soil conservation measures.

The innovation of the present study is to explore the impact of soil erosion on total and minimum data sets derived soil qualities and their responses to a series of high resolution topographic indices from unmanned aerial vehicle. Therefore, the aims of this study were to (i) recognize soil erosion characteristics along the fluctuated slope in an agricultural land, (ii) identify the variation of soil quality on the slope, and (iii) disclose the impacts of soil erosion and slope topography on soil quality in the black soil region, northeastern China.

## Materials and methods

### Study site

**Figure 1:**
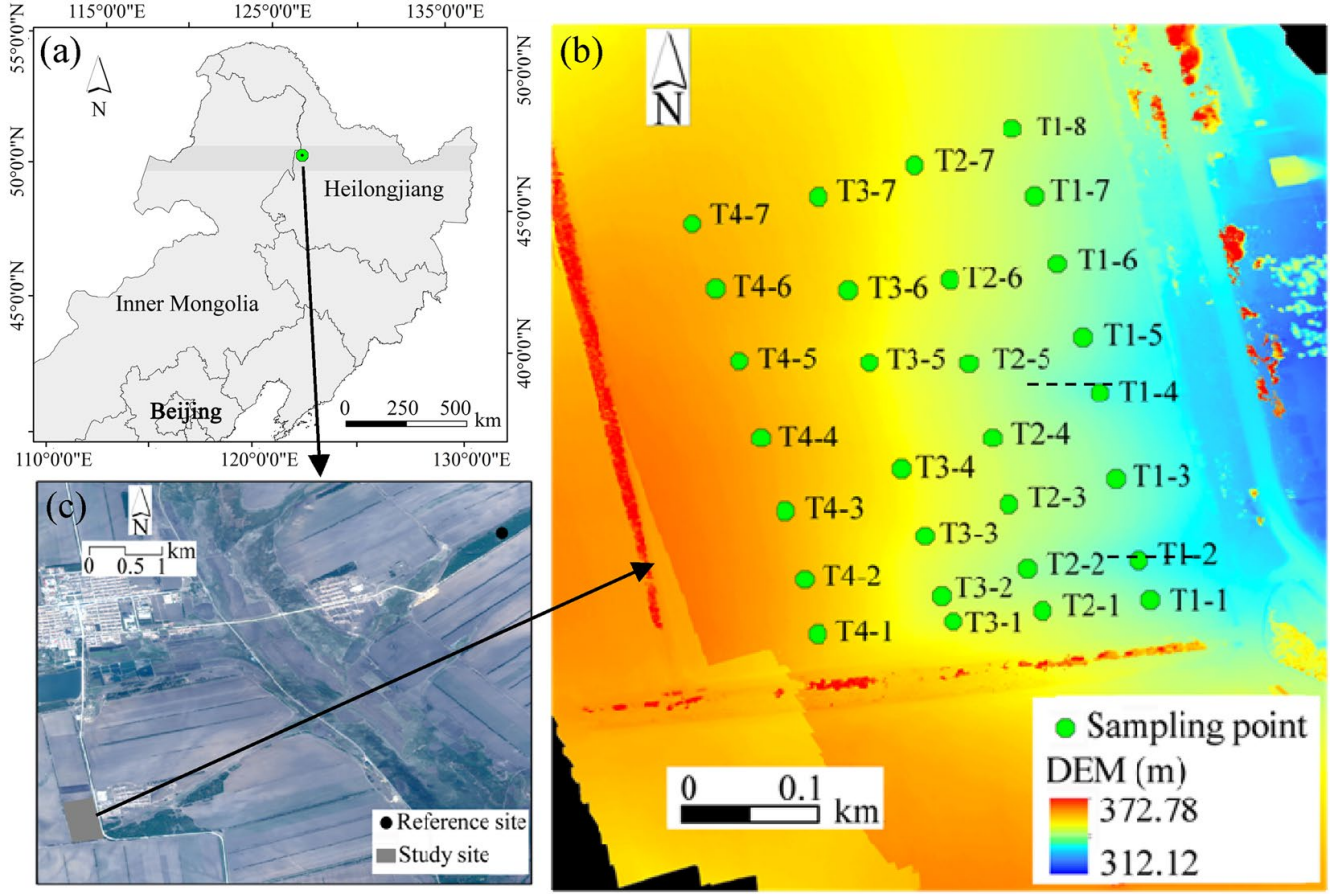
The locations of the ^137^Cs reference sampling site (**a**,**b**) and the sampling points (**c**) in an agricultural field. The sampling points along the transects "Introduction"–"Discussion" were indicated with a transect name and a number. The red lines at the edge of the digital elevation model (DEM) are farmland shelterbelts. *Note* the dotted line in the field represents developed ephemeral gullies. Figure 1 was created using ArcGIS 10.5 software at https://soft.wxqilinz.cn/gis-jb61d/.

Gentle and long slopes characterize the study region. As a representative farmland, an agricultural field in the Heshan Farm, northwestern Heilongjiang Province, China (125° 11′ E, 48° 56′ E;) was selected in the present study (Fig. 1). The field covers an area of around 25 ha. The elevations of the field range from 312 to 372 m. The slope is longer than 500 m, and the slope degrees range from 0.50 to 4.12°.

The mean precipitation was 530 mm, 75% of which falls between June and September during 1960–2020. The soil is classified as Udic Argibbrorll in the USDA Taxonomy, the soil parent material is Quaternary lacustrine and fluvial sand beds or loess sediment. The main texture classes of the soil are silty clay that is susceptible to soil erosion. In the field, except for sheet erosion, ephemeral gullies also exist (the dashed lines in Fig. 1c).

The study region has been cultivated for farmland since the 1950s and the environmental conditions have not changed over the past decades which generally coincide with the time of ^137^Cs deposition (i.e., 1954). In the study region, the main crops are spring wheat, corn, and soybean, which are grown in rotation. A single tillage operation is used with a cultivation depth of ca. 25 cm in late autumn. Farmland shelterbelts were constructed around the field, and the tillage direction is usually parallel to the shelterbelts.

### Soil sampling

Sampling was undertaken in late October 2021 when the crop was harvested. In order to reflect the impact of slope morphology on soil erosion and soil quality, four transects (i.e., transects "Introduction"–"Discussion" in Fig. 1c) were selected along the direction of shelterbelts, and a 5.5-cm diameter hand core sampler was used to collect the samples. The soil cores were taken to a depth of around 40 cm on the eroded area, and 60–120 cm depth at the deposition sites to ensure that all the cores collected the full depth of the ^137^Cs profile. The distance between the sampling points along each transect was 40–80 m depending on slope morphology. In total, 29 soil samples along the four transects in the agricultural land were collected.

The background value of ^137^Cs inventory at the study site had already been determined by Fang et al.^32^. Therefore, soil samples at the reference site were not collected in the present study.

### Extraction of topographic factors

In late 2021, the Unmanned Aerial Vehicle images were acquired using *DJI Phantom 4* with a longitudinal overlap of 75%. The choice of this time was to derive actual ground surface images to obtain field topography because the crops in the field were harvested at this time. The true color images and digital elevation model (DEM) in 20-cm spatial resolution was obtained using Agisoft Photoscan v 1.2.4 software (Fig. 1b). The DEM was then imported into ArcGIS software to extract topographic factors. According to other researchers^12,41,42^, several topographic factors that are relevant for soil erosion were extracted, including slope gradient, slope length and slope gradient (LS) factor, slope curvature, convergence index, relative slope position, and topographical wetness index.

### Laboratory analysis

The samples were air-dried, weighted, and divided into three parts. One part was passed through a 2-mm sieve for ^137^Cs detection and soil texture measurement, one part was passed through a 0.15-mm sieve for measuring soil organic matter (SOM) and total nitrogen(TN) contents, and the rest was passed through a 0.10-mm sieve for pH, available phosphorous (AP), available potassium (AK), and cation exchange capacity (CEC) measurements. The radioactivity of ^137^Cs was measured by a hyper-pure coaxial Ge detector with a multichannel analyzer at a 662 keV peak and a counting time over 80,000 s. The SOM concentration was measured using a wet combustion method^33^. Soil TN content was determined by the Kjeldahl digestion method. Sediment size parameter was determined using a Malvern Mastersizer 2000 laser analyser. Soil pH was determined by potentiometric method^4^. CEC was measured using sodium acetate leaching method^36^. AP and AK were determined using the sodium bicarbonate method and flame atomic absorption spectrophotometry method, respectively.

### Soil erosion estimation

A number of approaches have been proposed to estimate soil erosion by measuring^137^Cs content in cultivated lands. In the present study, the widely used Mass Balance Model 2 (MBM2)^43^ was employed to estimate the rates of soil erosion and gain, and the site specific parameters $\gamma$, *H*, and d in the model were given values of 0.6, 4 kg m^−2^, and 312 kg m^−2^ according to our previous study^34^.

The ^137^Cs reference site was around 5 km away from the study agricultural land (Fig. 1). The reference baseline of ^137^Cs inventory (i.e., 2506 Bq km^−2^) had been given in previous study by Fang et al.^32^. Because ^137^Cs can decay with time, the current baseline value of ^137^Cs (i.e., 1946 Bq km^−2^) was then obtained by using the measured reference baseline in 2010, its half-life (i.e., 30.2 years), and the elapsed time (Eqs. 1 and 2).

1 $$\text{N}\left(\text{t}\right)={N}_{0}{e}^{-\lambda {t}^{\prime}}$$

2 $$\uplambda =\frac{ln2}{T}$$

where N(t) is the ^137^Cs inventory at time t, N_0_ is the original ^137^Cs inventory, ${t}^{\prime}$ is the elapsed time (i.e., 11 years), T is the half-life of ^137^Cs tracer.

In order to run MBM2, the measured ^137^Cs contents (Bq kg^−1^) were further converted into their inventories (Bq m^−2^) by using the total weight of each sample and the cross sectional area of the sampler.

### Soil quality assessment

#### Determination of the minimum data set

The minimum data set (MDS) is usually used to represent the soil quality information of the total data set (TDS) using principal component analysis (PCA). The soil indicators were first standardized and then were processed through PCA using SPSS software. Only the principal components (PCs)with the eigenvalues ≥ 1.00 were kept (Table 1).Then, the soil indicators with factor loading ≥ 0.5 were labeled. If the factor loading of one indicator was ≥ 0.5 that appeared in more than one PC, it was assigned into the PC within which the soil indicator was lower correlated with other indicators. For those soil indicators kept in one PC, correlation analysis was conducted (Table 2). Therefore, only SOM in PC1 was entered into MDS. Correlation analysis for the four soil indicators sand, silt, pH and CEC in PC2 indicated that CEC can first enter into MDS, and then sand indicator was also selected in the MDS according to the correlation matrix among the factors and higher *Norm* values that reflect each indicator’s capacity to explain the comprehensive information of the TDS. The *Norm* value was calculated by using Eq. (3):

3 $${Norm}_{ik}= \sqrt{\sum_{\text{k}=1}^{\text{i}=1}{\text{u}}_{\text{ik}}^{2}{\uplambda }_{\text{k}}}$$

where the *Norm* value is the *i*th variable in the *k* PCs with eigenvalues ≥ 1.00, U_ik_ is the variable loading in the *k*th PC, and ${\uplambda }_{\text{k}}$ is the engenvalue of the *k*th PC.

**Table 1:** Component matrix of principal component analysis (PCA), *norm* values of soil indicators, and the minimum data set (MDS).

Soil indicator	Principal component		Norm	MDS
	PC1	PC2		
Clay	**0.791**	− 0.283	1.525	–
Silt	− 0.403	− **0.733**	1.232	–
Sand	− **0.668**	**0.660**	1.526	Sand
AP	**0.743**	0.086	1.393	–
CEC	0.366	0.497	0.951	CEC
AK	**0.804**	0.055	1.504	–
PH	0.175	**0.615**	0.881	–
SOM	**0.857**	0.098	1.607	SOM
TN	**0.567**	0.267	1.118	–
Eigenvalue	3.492	1.770	–	–
% of variance	38.805	38.805	–	–
Cumulative (%)	19.603	58.468	–	–

The bold values were the initially kept soil indicators in the PCs with eigenvalue ≥ 1.00.

**Table 2:** Pearson correlation matrix between ^137^Cs inventories and soil properties.

	^137^Cs	Clay	Silt	Sand	AP	CEC	AK	pH	SOM
Clay	0.474**	1							
Silt	− 0.151	− 0.083	1						
Sand	− 0.330	− 0.829**	− 0.489**	1					
AP	0.452*	0.424*	− 0.013	− 0.364	1				
CEC	0.488**	0.170	− 0.183	− 0.046	0.211	1			
AK	0.434*	0.514**	− 0.095	− 0.397*	0.546**	0.207			
pH	0.317	0.007	− 0.214	0.114	0.224	0.347	0.054		
SOM	0.705**	0.586**	− 0.156	− 0.426*	0.634**	0.274	0.647**	0.084	
TN	0.514**	0.211	− 0.077	− 0.141	0.305	0.211	0.501**	0.154	0.485**

* and ** indicates the correlation coefficients were significant at 0.05 and 0.01 levels.

#### Calculation of soil quality index

The soil quality index (SQI) is a widely used method to measure soil quality, and can be obtained by multiplying the weight and score of each soil indicator (Eq. 4):

4 $${\text{SQI}} = \sum\limits_{{{\text{i}} = 1}}^{{\text{n}}} {{\text{w}}_{{{\text{xi}}}} {\text{f}}_{{{\text{xi}}}} }$$

where *i* is the number of soil indicator, and w_xi_ and f_xi_ are the weight of and the score of the x_i_ soil indicator.

In the present study, the score of soil indicator was obtained by three standard scoring functions, i.e., the “more is better (MB)” equation, the “less is better (LB)” equation, and the “optimal range (O)” equation. The equations were calculated by using Eqs. (5)–(7):

5 $$\text{MB}:\text{ f}\left(\text{x}\right)=\left\{\begin{array}{c}0.1 \,\,\,\,\,\,\,\,x\le a\\ 0.9\times \frac{(\text{x}-\text{a})}{\text{b}-\text{a}} \,\,\,\,\,\,\,\,a<x<b\\ 1 \,\,\,\,\,\,\,\,x\ge b\end{array}\right.$$

6 $$\text{LB}:\text{ f}\left(\text{x}\right)=\left\{\begin{array}{c}1.0 \,\,\,\,\,\,\,x\le a\\ 0.9\times \frac{(\text{b}-\text{x})}{{\text{x}}_{\text{max}}-{\text{x}}_{\text{min}}} \,\,\,\,\,\,\,a<x<b\\ 0.1 \,\,\,\,\,\,\,\,\,x\ge b\end{array}\right.$$

7 $$\text{O}:\text{f}\left(\text{x}\right)=\left\{\begin{array}{c}0.1 \,\,\,\,\,\,\,\,\,x\le {\text{a}}_{1} or x\ge {\text{b}}_{2}\\ 0.9\times \frac{\text{x}-{\text{a}}_{1}}{{\text{a}}_{2}-{\text{a}}_{1}}{\text{a}}_{1}<x<{\text{a}}_{2}\\ 1 \,\,\,\,\,\,\,\,{\text{ a}}_{2}\le x\le {\text{b}}_{1}\\ 0.9\times \frac{\text{x}-{\text{b}}_{1}}{{\text{b}}_{2}-{\text{b}}_{1}}{\text{b}}_{1}<x<{\text{b}}_{2}\end{array}\right.$$

where f(x) is the score of soil indicator between 0 and 1, x is the variable value, and “a” and “b” are the minimum and maximum values, respectively.

According to previous studies^44,45^, the scores of clay content, silt content, CEC, AK, SOM, and TN were obtained through BM equation, the sand content score was derived from LM equation. The “Optimum is better” scoring system was used to soil pH. Since there is no threshold value in literature for soil pH, the threshold of 7 was given. When soil pH is less than 7, OM equation was used, and when pH is larger than 7, the LM equation was employed.

The weights of soil indicators were obtained through the ratio of community for each soil indicator to the sum of communities for all the soil indicators through PCA analysis^2^. Therefore, the weights of soil indicators in TDS and MDS were obtained (Table 3). Then the SQIs at the sampling sites were calculated using Eq. (2).

**Table 3:** Estimated communality and weight values of each soil indicator in the TDS and MDS in the agricultural land.

Soil indicator	TDS		MDS	
	Communality	Weight	Communality	Weight
Clay	0.706	0.134	–	–
Silt	0.539	0.102	–	–
Sand	0.882	0.168	0.538	0.352
AP	0.559	0.106	–	–
CEC	0.381	0.072	0.258	0.169
AK	0.650	0.124	–	–
pH	0.409	0.078	–	–
SOM	0.744	0.141	0.732	0.479
TN	0.393	0.075	–	–

### Data analysis and treatment

The datasets were statistically analyzed using SPSS 14.0 software to conduct PCA, Pearson’s correlation matrix, and the least significant difference (LSD) analysis. Spatial distribution maps of soil erosion and soil quality were made using ArcGIS 10.5 software through the kriging interpolation method. Topographic factors were extracted using SAGA 7.6.2 software. Statistic figures were made using Origin 15.0 software.

## Results

### ^*137*^*Cs inventories and soil properties*

The ^137^Cs inventories for the 29 soil cores collected from the agricultural field varied greatly. The ^137^Cs inventories ranged from 1067.28 to 2623.01 Bq m^−2^, with an average of 1751.08 Bq m^−2^and a standard error of 389.62 Bq m^−2^. A majority (72.4%) of the soil samples had lower values than the reference value.

Soil properties also varied greatly (Table 4). The soil texture of the samples is silty, with mean silt content percentage of 64.00%, and mean sand and clay content percentages occupied 18.56% and 17.44%, respectively. The mean content of soil AP is 38.82 mg kg^−1^, ranging from 22.40 to 55.90 mg kg^−1^with a standard error of 8.45 mg kg^−1^. The soil had higher AK contents, with an average of 189.76 mg kg^−1^, ranging from 138 to 232 mg kg^−1^. The mean values of soil CEC were 12.96 cmol kg^−1^, ranging from 11.30 to 14.80 cmol kg^−1^ with a standard error of 0.87 cmol kg^−1^. The black soil had high SOM content, with an average of 57.60 g kg^−1^. Soil TN content ranged from 0.17 to 0.22%, with an average of 0.19%.The soils of the land were neutral to slightly acidic. The soil pH ranged from 5.8 to 7.3, with a mean value of 6.68 and a smaller standard error of 0.48.

**Table 4:** Statistics of ^137^Cs inventories and soil properties.

Soil indicator	Unit	Minimum	Maximum	Mean	St.d Error
^137^Cs inventory	Bq m^−2^	1067.28	2623.08	1751.09	389.62
Clay	%	12.49	21.67	17.44	2.64
Silt	%	60.65	67.87	64.00	1.70
Sand	%	13.69	24.01	18.56	3.02
AP	mg kg^−1^	22.40	55.90	38.82	8.45
CEC	cmol kg^−1^	11.30	14.80	12.96	0.87
AK	mg kg^−1^	138.00	232.00	189.76	24.85
SOM	g kg^−1^	30.30	57.60	42.18	6.99
TN	%	0.17	0.22	0.19	0.02
pH	–	5.80	7.30	6.68	0.48

### Soil *erosion*

Soil erosion (“ − ” sign) and gain (“ + ” sign) rates were given in Table 5. In the study agricultural land, the mean soil erosion rate was 275 t km^−2^ yr^−1^.The largest soil erosion rate was 1870 t km^−2^ yr^−1^, and the maximum gain rate was 1557 t km^−2^ yr^−1^, with a standard error of 838 t km^−2^ yr^−1^. Around 72.4% of the sample sites suffered soil erosion, and the mean soil erosion rate of 685 t km^−2^ yr^−1^ in erosion area was significantly lower than that (i.e., 517 t km^−2^ yr^−1^) in the deposition area.

**Table 5:** Soil loss and gain rates for the sampling points in the agricultural field.

	Gross samples	Samples from erosion area	Samples from deposition area
Min. (t km^−2^ yr^−1^)	− 1870	− 1870	117
Max. (t km^−2^ yr^−1^)	1557	− 20	1557
Mean(t km^−2^ yr^−1^)	− 275 (a)	− 685(b)	801(c)
Std. (t km^−2^ yr^−1^)	838	501	517
Skewness	0.42	− 0.82	− 0.04
Number of samples	29	21	8

**Figure 2:**
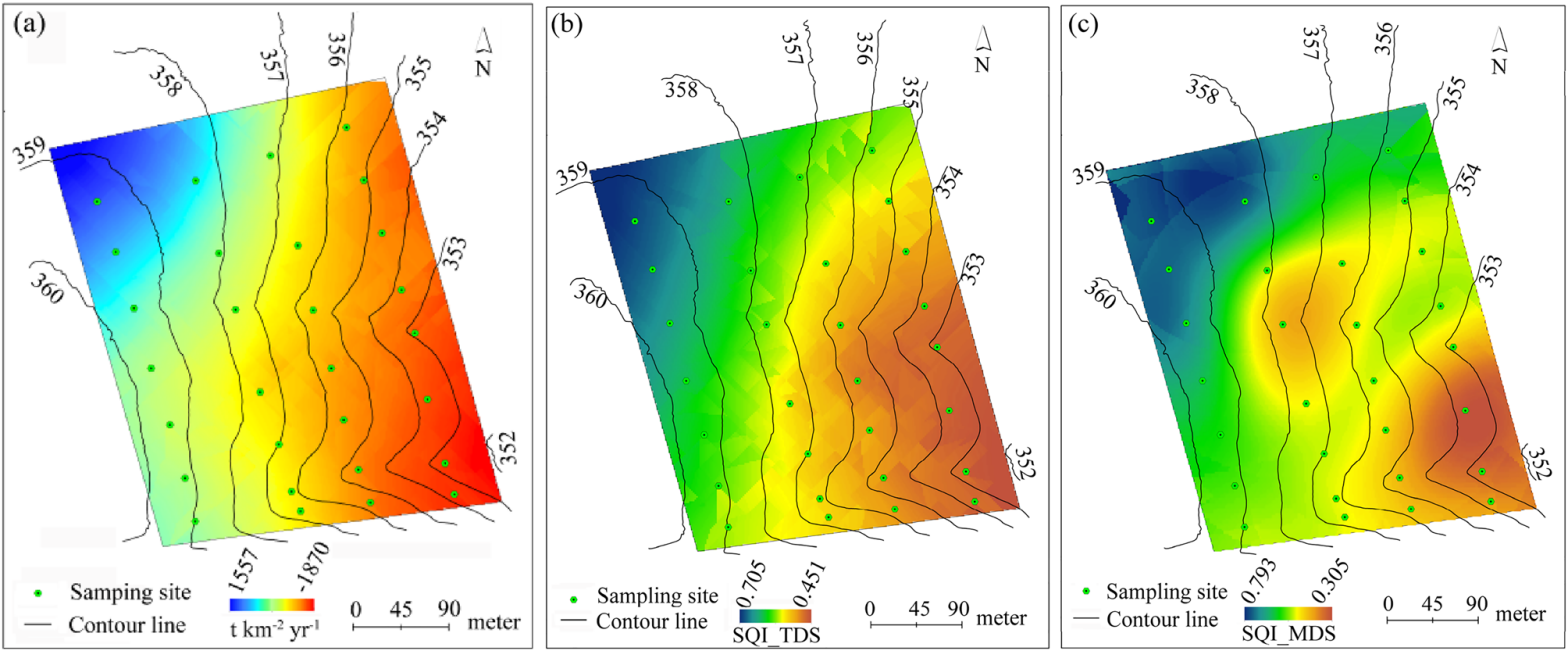
Spatial distributions of (**a**) soil erosion and gain rates, (**b**) soil quality index derived from total data set (SQI_TDS), and (**c**) soil quality index derived from the minimum data set (SQI_MDS).

Figure 2a depicted a spatial pattern of soil erosion and gain in the study field derived from kriging interpolation of the sampling sites. Soil erosion mainly occurred in the lower part of the field where ephemeral gullies developed. However, soil gain occurred in its upper part which agreed with the field topography (Fig. 1).

### Soil quality

**Figure 3:**
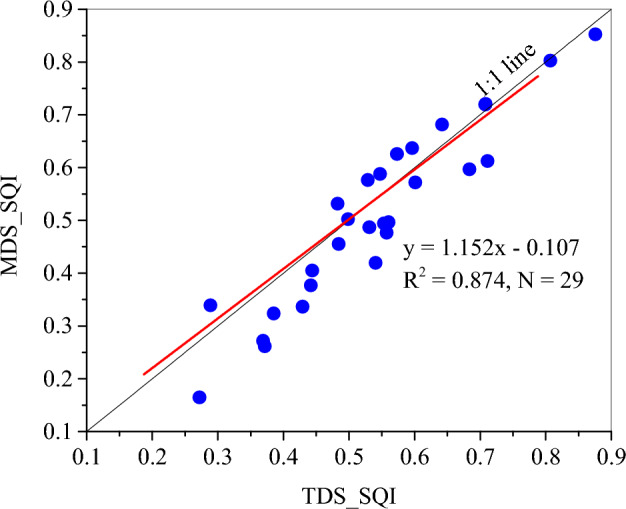
Relationship between SQI_TDS and SQI_MDS derived from the sampling sites.

Based on the scores of soil indicators and their weights, both TDS and MDS derived SQIs (i.e., SQI_TDS and SQI_MDS) at the sampling sites were obtained. SQI_TDS ranged from 0.270 to 0.880, with an average of 0.551 and a standard error of 0.149. In comparison, SQI_MDS ranged from 0.160 to 0.950, and had a comparable mean value of 0.527 to that of SQI_MDS (Table 6). The SQI_TDS values were significantly correlated with SQI_MDS values, with a determinant coefficient of 0.874 (Fig. 3).

**Table 6:** Statistics of the SQIs derived from TDS and MDS for the sampling points.

	Min	Max	Mean	St.D	Skewness
SQI_TDS	0.270	0.880	0.551a	0.149	0.200
SQI_MDS	0.160	0.950	0.527a	0.183	0.248

Spatially, the two sets of SQIs had similar distribution patterns. Upper agricultural field had higher SQI, and lower part had lower SQI. Both SQI_TDS and SQI_MDS spatial distributions were similar to that of soil erosion and gain rates (Fig. 2).

### Relations soil quality and soil erosion

**Figure 4:**
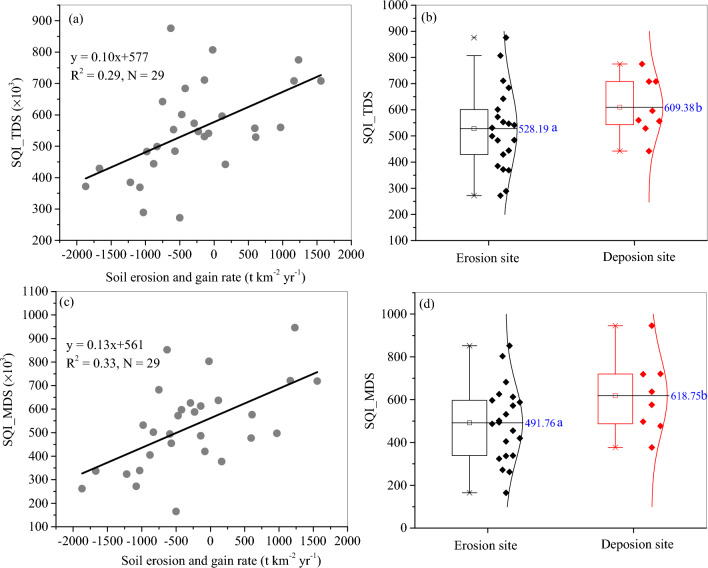
Relationships between soil erosion and SQI_TDS (**a**) and SQI_MDS (**c**), and the values of SQI_TDS (**b**) and SQI_MDS (**d**) at the erosion and deposition sites. Different letters after a number indicated a significant difference between SQIs at different sites (LSD test, *p* < 0.05).

Figure 4a and c demonstrated that both SQI_TDS and SQI_MDS increased with increasing soil gain rates (minus and positive signs indicating soil erosion and soil gain, respectively), and can be linearly determined by soil erosion and soil gain rates. The SQI_TDS and SQI_TDS values at the erosion sites were significantly lower than those at the deposition area. F- and t-tests demonstrated that both the equations and the regression coefficients are significant at the 0.01 level although the determination coefficients were not high (Fig. 4ac).

Spatially, the distribution patterns of SQI_TDS and SQI_MDS in the agricultural field were almost the same to that of soil erosion (Fig. 2). The highest SQI-TDS and SQI-MDS of 0.71 and 0.72 existed in the upper-left of the field and the lowest values of 0.27 and 0.16 for SQI-TDS and SQI-MDS in the lower-right part of the field, implying that SQI is greatly influenced by soil erosion in the black soil region.

## Discussion

The MDS is widely used to evaluate soil quality because TDS can take much more time and cost more to estimate soil quality^17,46^. According to the global research results, most of the used soil indicators in the present study were among the commonly used factors for evaluating soil quality^4,47–49^^.^ Therefore, soil quality of the black soil can be well evaluated using the TDS. Some information is lost when MDS was used. However, SQI_MDS can explain 87.4% information of SQI_TDS although only using three soil indicators (i.e., SOM, CEC, and sand content) in the MDS. Therefore, instead of using TDS to estimate soil quality, the MDS determined by PCA and correlation coefficient method has been widely used in the world^4,7,49^ and can be a successful alternative for soil quality assessment in the black soil region. Furthermore, the value range of the SQI_MDS is higher and seems to better separate the soil quality (Fig. 2c). This finding was also reported by other studies^46,50^.

Derakhshan-Babaei et al.^12^ pointed out that SOM and sand content play a more vital role than other soil properties when soil quality was assessed. The present study quite agrees with this conclusion. This is because SOM affects most other soil properties, and the increase in SOM can improve other soil properties, resulting in improved soil quality^51^. Soil CEC represents the ability to hold positively charged irons, and higher CEC can also be attributed to higher SOM and lower pH^52,53^. AP and AK are crucial soil indicators supplying nutrients for plant growth^54^. SOM and TN are two important nutrients relating to biogeochemical cycling^55^. However, they are preferentially removed by soil erosion^56^. The reduction of SOM in soils can negatively affect some physical and biological properties such as aggregate stability, soil bulk density, soil water infiltration, and soil microbial activity et al., resulting in reduced soil quality, crop production and environment quality^57^. Sand content also plays an important role in influencing soil quality in the present study. Samaei et al.^17^ also pointed out that high gravel contents in northeastern Iran had the most limitation for soil quality. In the study region, the weight of sand content was higher (i.e., 0.352) in the MDS (Table 3), indicating its important role in determining soil quality.

Soil erosion directly affects soil quality due to poor land use practices and management, and the SOM and soil nutrients decrease with increasing soil erosion^1^. In the study field, the annual soil loss rate during the past 50 years was − 275 t km^−2^ yr^−1^, resulting from both water and tillage erosion. Thaler et al.^58^ demonstrated that water erosion is dominant in areas with steep and concave slopes whereas erosion on upland convex hilltops is dominated by tillage. In the corn field in US, around 30% and 70% of the observed B-horizon exposure occurred on concave and convex topography. The loss of the top soil layer with rich SOM and soil nutrients could thus greatly decrease soil quality (Figs. 2 and 4), resulting in reduced soil productivity of the black soils^39,40^.In the purple soil region, southwestern China, Jin et al. (2021) also concluded that soil productivity presented a decreasing trend with increasing soil erosion. Using plot data, Mandal et al.^1^ demonstrated that SQI decreased with the increase in phases of soil erosion. Therefore, the intense soil erosion with ephemeral gullies in the lower field can explain lower soil quality, and the redeposited soil with rich SOM and nutrients behind the shelterbelts led to higher SQI values (Fig. 4b, d).

Soil erosion and soil properties are greatly affected by topographic factors such as slope and slope positions^59^. Steep slope usually induces higher soil erosion. However, the gentle slope in the study region did not significantly affect soil erosion, as pointed out by Fang et al.^32^. Furthermore, except for the relative slope position RSP, other topographic factors also did not significantly affect soil erosion and soil quality (Table 7), implying that soil erosion types are more important in influencing soil erosion and soil quality (Fig. 1). The developed ephemeral gullies could greatly affect the function of topographic factors^60^. In contrast, the upper field is just behind shelterbelts, where water flow energy is weak leading to less soil erosion and sediment to be redeposited. This inference has been found by Fang et al.^32^. Therefore, the innovations of the present work have at least two aspects. One aspect is that the contribution of soil erosion in influencing soil quality was given using both SQI-TDS and SQI-MDS. The other aspect is that the changes of soil erosion and soil quality with varying land morphology and the main topographic factor were pointed out using the high-resolution DEM derived from unmanned aerial vehicle.

**Table 7:** Pearson correlation coefficients among soil erosion intensity (SEI), soil quality index, and topographical factors.

	SEI	SQI_TDS	SQI_MDS	TWI	Cur	LS	RSP	S	L
SQITDS	0.544**								
SQIMDS	0.576**	0.938**							
TWI	0.064	0.097	0.168						
Con	− 0.233	− 0.142	− 0.081	− 0.399*					
LS	− 0.162	− 0.175	− 0.160	0.442*	− 0.187				
RSP	0.444*	0.149	0.155	− .402*	0.121	− 0.213			
S	− 0.231	− 0.318	− 0.381*	− 0.326	0.162	0.431*	0.028		
L	0.281	0.194	0.336	.575**	− 0.097	0.281	− 0.154	− 0.036	
Cur	− 0.031	0.033	0.116	− 0.050	0.302	0.293	0.047	0.127	0.170

TWI is topographic wetness index, Cur is curvature, LS is USLE slope length and slope factors, RSP is the relative slope position , S is slope degree, and L is slope length, and Cur is curvature index.

* and ** indicates the correlation coefficients were significant at 0.05 and 0.01 levels.

In the black soil region, soil erosion usually occurs on the upper slope, and soil gains on the lower slope^60,61^. However, in the present study, around 72.4% of the sampling sites suffered soil erosion in the field. This could be because samples were not collected near the lower field edge where soil deposition could occur. This inference would have been verified by severe sediment deposition along the field edge of a small catchment in the study region^32^. In the present study, only 29 sampling sites were done that could to some extent limit the yielded results. Furthermore, biological indicators that can also reflect soil quality^62^ were not included in the present study. Therefore, more soil samples and more biological indictor analysis in different places can better reflect soil redistribution pattern to improve soil quality assessment. However, in the present study, the MDS derived SQI can still better reflect soil quality and its response to soil erosion in the black soil region.

## Conclusions

In the agricultural field, northeastern China, the soil erosion rates ranged from − 1870 to 1557 t km^−2^ yr^−1^ with an average of − 275 t km^−2^ yr^−1^. Three soil indicators (i.e., SOM, ECE, and pH) entered the MDS. Soil quality was greatly affected by soil erosion, with Adjust R^2^ of 0.29 and 0.33 for SQI_TDS and SQI_MDS, respectively. Both soil erosion and soil quality are affected by field topographical characteristics, especially by the index of relative slope position.

More soil samples and more biological indictor analysis could better improve soil quality assessment. However, this study can still extend our insight into erosion induced land degradation in the world.

## Data Availability

The datasets used and/or analysed during the current study available from the corresponding author on reasonable request.
